# Reduction of reactive red 241 by oxygen insensitive azoreductase purified from a novel strain *Staphylococcus* KU898286

**DOI:** 10.1371/journal.pone.0175551

**Published:** 2017-05-03

**Authors:** Numrah Nisar, Amber Aleem, Faiza Saleem, Fakhra Aslam, Ammara Shahid, Hina Chaudhry, Kausar Malik, Abdulhadi Albaser, Amjad Iqbal, Rashad Qadri, Yaodong Yang

**Affiliations:** 1 Department of Environmental Sciences, Lahore College for Women University Lahore, Lahore, Pakistan; 2 Department of Biotechnology, Lahore College for Women University Lahore, Lahore, Pakistan; 3 Department of ORIC, Lahore College for Women University Lahore, Lahore, Pakistan; 4 Faculty of Sciences, University of Sebha, Sebha, Libya; 5 Hainan Key Laboratory of Tropical Oil Crops Biology/Coconut Research Institute, Chinese Academy of Tropical Agricultural Science, Wenchang, Hainan, China; 6 Department of Agriculture, Abdul Wali Khan University Mardan, Mardan, Pakistan; 7 Institute of Horticultural Sciences, University of Faisalabad, Faisalabad, Pakistan; Lee Kong Chian School of Medicine, SINGAPORE

## Abstract

An oxygen insensitive azoreductase was purified from a novel bacterial strain (*Staphylococcus* sp. KU898286) that was isolated from an abandoned site of the textile waste discharge unit. The isolated enzyme had efficiently cleaved the azo-bonds through reductive transformation under aerobic conditions. Initial phenotypic characterization and final construction of phylogenetic tree on the basis of 16s rDNA demonstrated 99% resemblance of the isolate to *Staphylococcus aureus*. The purified azoreductase was found to have a broad spectrum activity that reduced RR241 at a concentration of 50mg/L with pH between 6–8 and 30°C temperature). Besides, the reactive red 241 (RR241) was reduced at extracellular level as well as NADH dependent intracellular level. Complete reduction/ decolourization of RR241 were achieved after 18 hrs of exposure. The final degradation product observed to be 2-nephthol was purified by High Pressure Liquid Chromatography (HPLC) and the molecular mass was computed by Gas Chromatography-Mass spectroscopy (GC-MS). The study revealed a cost effective and eco-friendly approach to degrade the toxic dyes into less toxic products by *Staphylococcus* sp. KU898286.

## Introduction

Azodyes are the class of textile dyes, characterized by -N = N- azobonds attached to the aromatic ring. The dyes are extensively used in the textile industry because of their brilliant color shades, high wet fastness profiles with ease of application and minimal consumption of energy [1–3]. Consequently, recent research is highly focused towards the utilization of efficient, eco-friendly and cost-effective biological treatments to decolourize degrade and detoxify dye contaminated effluents [4, 5]. The general approach of this biological treatments or bioremediation is to improve the natural degradation capacity of the indigenous microorganism [1, 5]. Bioremediation of these azodyes causes mineralization of these compounds into simpler inorganic substitutes, which are not toxic to the existing life forms. The basic concept involved in the decolourization and degradation of azodyes is the breakdown of azo-bonds, leading to the removal of color [6]. A number of bacterial species belonging to genera *Enterococcus*, *Rhodobacter* and *Clostridium* have been isolated so far to bio-treat textile dyes [7–10]. The associated enzymes under aerobic conditions have the ability to mineralize azo-compounds into simpler inorganic substitutes. Initially azo-compounds are reduced to their corresponding amines (e.g. generation of nitroanilines) followed by the cleavage of the azo-linkages (-N = N-). This exposes the sites, which favours in further reduction of the dyes until complete mineralization [2, 11]. In the current study, a novel strain was isolated from a textile sludge site and characterised for its efficient degradation of RR241. The enzymes involved were purified and the products of degradation were identified.

## Material and methods

### Dyes and chemicals

Highest purity reagent grade chemicals were used for the study. RR241, all bacterial growth mediums and bovine albumin serum (BSA) was purchased from Sigma-Aldrich, UK. Sodium chloride, and anhydrous Na_2_SO_4_ were purchased from Merck, Germany. The stock solution (100mg/L) of reactive red 241 was prepared using deionized water and working solution obtained via serial dilution.

No specific permission was required for any activity of the presented research.

### Isolation and selection of effective dye degrading strain

Soil sample as described by Kalyani et al., [12] was collected from an abandoned site of a textile industry located in the suburbs of Lahore (coordinates of the area, DMS latitude 31° 34ʹ 55.3620ʺ N; longitude 74° 19ʹ 45.7536ʺ E) in March, 2016. About 1g of of soil was placed into M9 medium (10mL) with RR241 (50μg/mL) as sole carbon source and left in the dark at room temperature for about 2 weeks. About 1 mL of solution was removed after agitation and placed into fresh M9 medium amended with RR241 (50μg/mL) and incubated for further 2 weeks. The process was repeated six times and finally dilutions were made and approx. 1ml of enrichment culture was streaked onto nutrient agar plates (plates were made for each dilution) constituted with RR241(50μg/mL) and incubated overnight at 30°C. Metabolizing colonies (showing decolourization) were isolated and maintained in M9 media plates followed by the generation of glycerol stocks (40%) from 16hr culture of isolates individually in M9 media. To determine the decolourisation capacity and toxicity assessment, colonies with the clear zones were inoculated in the mineral salt medium (MSM) agar plates (K_2_HPO_4_ 1.73 gL^–1^; FeSO_4_ 7H_2_O 0.03 gL^–1^; KH_2_PO_4_ 0.68 gL^–1^; peptone 1.0 gL^–1^; MgSO_4_·7H_2_O 0.1 gL^–1^; NaCl 0.1 gL^–1^; NH_4_NO_3_ 1.0 gL^–1^; CaCl_2_·2H_2_O 0.02 gL^–1^) with varied concentration of RR241 (0–100 mg/L) at different pH (2–12). Bacterial strain with the maximum decolourisation ability was selected.

The exponential phase bacterial cells (OD_600nm_) of the isolates were harvested by centrifugation at 6000×g for 5 min at 4°C to prepare the resting cells. These cells were then washed severally with MSM before storing at 4°C. A 100 mL of Difco Sporulation medium (DSM) amended with RR241 (0 – 200mg/L) at a constant concentration of resting cells (100μl/100ml of media) was employed to analyse the degradation and decolourisation rate using a UV–visible spectrophotometer at λ_max_ = 541. For this purpose, 2 mL of the aliquot was withdrawn from the culture media at regular intervals of 0–48 h and the decolourisation percentage (% D) calculated as follows:

$$\%\;D=\frac{Initial\;abs.-Final\;abs.}{Initial\;abs.}\times 100$$ (1)

### Morphological and phylogenetic characterisation of bacterial strain

The most efficient strain (S1) exhibiting the highest decolourisation potential was analysed for its morphology and physico-biochemical properties. The estimations of colony shape, size and gram staining were carried out initially. Further tests such as catalase, oxidase, urease, haemolytic activity, motility, indole, hydrolysis of indole and DNase were performed following standard procedures [13] to give a complete physico-chemical evaluation of the isolates. These tests were then evaluated according to Bergey’s manual [14] to estimate the physico-chemical properties of the strain. Final identification was conducted through 16S rRNA sequence determination. For this overnight culture (of isolated bacteria) was centrifuged (13000*g* for 5min) and the resulting pellet was washed thrice with milliQ water (×1) with final re-suspension was accomplished in 15μl milliQ water followed by boiling at 95°C for 5 min. Sample was cooled (on ice), spun (13000*g*) for 5 min and finally the supernatant was collected. Gene was then amplified by PCR using dNTPs (200μM each), GoTaq DNA polymerase, PCR buffer soln. (Promega) and primers AMP_F (5': GAG AGT TTG ATY CTG GCT CAG; Tm = 60.5) and AMP_R (5': AAG GAG GTG ATC CAR CCG CA; Tm = 68.9). PCR cycle consisted of initial denaturation at 95°C for 2 min (1 cycle); denaturation at 95°C for 30 s (30 cycles); annealing at 55°C for 30 s (20 cycles); extension at 72°C for 1 min 30 s (20 cycles); final extension at 72°C for 1 min (1 cycle). The amplicon (PCR product; 1500 bp) was analysed on 1% agarose gel and purified by PCr purification kit (QIAquick; Qiagen). PCR product was quanitified by a Nandrop 2000 spectrometer (Thermo scientific) and sequenced with both the selected primers (AMP_F and AMP_R; Macrogen Inc.). Seqman was employed to esemble the sequences and create a contig, which was searched on RDP database (https://rdp.cme.msu.edu). After custom sequencing by Macrigen Inc. the quality-trimmed extended contigs were generated by SPAdes programme, 3.6.1vs [15]. After removing gaps and analysing highly variable positions, a phylogenetic tree was generated from these contigs using Phylogeny.fr, 2016.

### Phylogenetic analysis

The DNA extracted from the harvested bacterial biomass (isolated strain) and its 16s rDNA was amplified and sequenced by universal forward primer: 5’-AGA GTT TGA TCC TGG TCA GAA CGC T-3’ and reverse primer ‘5-TAC GGC TAC CTT GTT ACG ACT TCA CCC C-3’. After removing gaps and analysing highly variable positions a phylogenetic tree was generated.

### Extraction of enzyme and gel electrophoresis

The resting cells from strain S1 were grown at 30°C in liquid MSM medium for 24 hrs to obtain a mid-log phase. About 10 ml of sample was taken from it and centrifuged for 15 min at 5000 rpm. Supernatant thus obtained was filtered by a sterile syringe filter (0.45 mm, Millipore). This supernatant was designated to be extracellular protein and was used against different concentration of RR241 to determine the degradation rate and products. To determine the intracellular activity of azoreductase, the pellet, thus obtained (after centrifugation) was sonicated (Sonics vibracell ultrasonic processor). In order to breakdown the cell wall and release the cytosolic proteins, the sonicator was set at 40 amps with 8 strokes per 4 seconds. The sonication was done for 2 min and the sample was cooled on ice before centrifugation. Sonicated sample was centrifuged (12,000×g, 20 min) and the supernatant containing crude enzyme was transferred to a fresh tube. The crude enzyme was then precipitated by addition of ammonium sulphate (80% saturation, 4°C). Precipitated enzyme was separated from the ammonium sulphate solution in the form of a pellet by centrifugation (10,000×g, 20 min). The pellet was further dissolved and dialyzed against 0.1 M phosphate buffer (pH 7.2) for 24 hrs at 4°C to remove low molecular weight impurities. The contents of the dialysis tubing were transferred to a tube, lyophyilized and kept frozen at -80°C till further use. The lyophilized enzyme was re-dissolved in 0.1 M phosphate buffer (pH 7.2) and applied to a sephadex G-100 column (1cm × 30 cm, Amersham Biosciences), previously equilibrated with the same buffer. The enzyme was eluted following linear gradient of 0–0.5 M with phosphate buffer saline (pH7.2, 0.5 M NaCl). About 2ml fractions were collected at a flow rate of 6 ml/ hr. Active fractions, thus obtained were pooled and lyophilized and was used as pure enzyme in further assays. The molecular mass of the protein was determined through polyacrylamide gel electrophoresis (SDS-PAGE analysis) and concentration of protein was analysed through standard protein assay: Bradford assay with Bovin albumin serum (BSA, Sigma Aldrich). The molecular weight of the enzyme was determined on 10% gel containing 0.1% SDS through the electrophoretic mobility of the enzyme with reference to DNA ladder and bovin serum albumin (66 KDa). The homogeneity was determined by PAGE carried out on 10% acrylamide gel without SDS. To determine the monomeric nature, polyacrylamide gel was run under native condition. The gel was stained with 150 μM of Navitan fast blue already made up in 0.1 mM phosphate buffer (pH 7) and then transferred to NADH (0.25mM) containing a phosphate buffer (pH 7) and incubated at 35°C. A clear, colourless area against dye background predicted the activity in the gel.

### Enzyme assay

The enzyme assay was determined by adding varying concentrations of RR241 in 1 ml cuvette placed at 1 cm path length in UV spectrometer. An amount of 100 μL of cell fraction, as prepared above, was added to each cuvette. Since the enzyme was rendered as NADH dependent, thus the reaction was initiated by the addition of 4–8 μL of NADH. A negative control was also run in parallel to the experiment with 0.1g/mL of lysozyme in replacement of enzyme to determine the oxidative reaction of RR241. The results were calculated through the disappearance of colour determined through absorption at maximum wavelength (λ_max_ = 541 nm). The results were recorded as a unit of enzyme activity, where one unit of enzyme activity was the enzyme required, under assay condition, to decolorize 1 μM of RR241 in one min.

### Localization of azoreductase activity

To localise the activity of azoreductase, cell free extract and resting cells were used for intracellular activity, whereas cell debris and the cell free homogenate was used to determine extracellular activities. The resting cells were prepared by harvesting exponential-phase bacterial cells followed by centrifugation (6000 × g at 4°C for 5 min). After centrifugation, the supernatant (homogenate) was removed and the pellet (resting cells, approx. 100 μg wet wt.) was washed thrice with MS medium. The pellet was then transferred to a 250 ml flask with 100 ml nutrient solution and 50 mg/L of RR241. The pH of the medium was set to pH 7 and aliquots were taken at constant intervals for 48 hrs. The reduction in RR241 was evaluated through determining wavelength at λ = 541nm. A similar experiment was repeated for homogenate (50 ml homogenate to 100 ml nutrient solution; 50 mg/L RR241 at pH 7), cell-free extract (2 ml extract to 100 ml nutrient solution; 50 mg/L RR241 at pH 7) and cell debris (100 μg wet wt in similar reaction mixture as described before). The cell free extract was prepared by growing cells in MN medium for 3 hrs. The cells were harvested by centrifugation (6000 × g at 4°C for 5 min) and then sonicated (Sonics vibracell ultrasonic processor). The cell debris was again removed by centrifugation (6000 × g at 4°C for 5 min). The pellet was referred as cell debris and supernatant was used as a cell free extract.

To determine NADH dependency, the reaction mixture contained about 20 mM potassium phosphate buffer (pH 7.2), 50 mg/L RR241, 0.5 mM of NADH and appropriate amount of reaction medium, i.e cell free extract, cell debris, homogenate or resting cells, so that the final volume is 2.5 ml. The reaction is stopped by boiling at 100°C for 5 min followed by centrifugation. The reduction was determined by measuring wavelength at λ = 541nm.

### Degradation of RR241 enzyme

The degradation products were evaluated by (HPLC) and Fourier Transformation Infra-Red spectroscopy (FTIR). For HPLC the supernatant, containing denatured enzyme (obtained through boiling the reaction mix), was extracted with an equal volume of ethyl acetate so that organic layer was separated from the dye component. The extract was then dried with anhydrous Na_2_SO_4_ and the final residues obtained were dissolved in 2 ml HPLC-grade methanol. The contents were then analyzed by HPLC, Waters 2690 instruments (Waters Corporation, UK), using C_18_ reversed phase column (symmetry 4.6–250 mm). The mobile phase was 60% acetonitrile and 40% water at a flow rate of 0.5 ml/min. For FTIR, the cell free supernatant was spread evenly on NaCl tablet and washed thrice with acetone. The analysis was conducted out on Perkin-Elmer 2000 FTIR spectrometer in the mid IR region of 400-4000cm^-1^ with a resolution 4cm^-1^ at 100 scans speed. The derivatization of metabolites was performed on QP2010 Shimadzu Gas chromatography equipped with Mass Spectroscopy (GC-MS). The assembly had a built-in autosampler and the sample was separated over BD-5 column (30 m × 0.25 mm × 0.25 mm). Helium was used as a carrier gas at a flow rate of 1 mL/min. The sample was dissolved in HPLC grade acetonitrile (3 mg/ mL) before injection into the port with help of microsyringe. The temperature of the injection port was kept at 250°C, where the holding temperature was 100°C and the detector temperature was kept to 260°C. The full scan acquisition was employed to analyse the peaks and comparison was made with standard run as well as MS analysis.

### Phytotoxicity assay

Phytotoxicity assay was performed to assess the toxicity of industrial effluent with RR241 before and after biodegradation by Staphylococcus sp. KU898286. To get this *S*. *vulgare* (wild type) and *A*. *thaliana* (Col 0) seeds (10 seeds each) were allowed to grow at under growth conditions of a photoperiod of 16/8h (L/D), temperature 23±2°C, RH 50/65% in compost (Levington’s F2 with sand and perlite; mixture of 4:1 organic compost) watered with 500mg/L of RR241 and biodegraded end product (recovered after completion of RR241 degradation by *Staphylococcus* sp. KU898286 by centrifugation and removal of cell debris). Germination (%), length of radicle and plumule was determined after 12 days of growth and evaluated against control seeds (watered with tap water).

## Results

### Morphological characterization of bacterial strains

**Fig 1:**
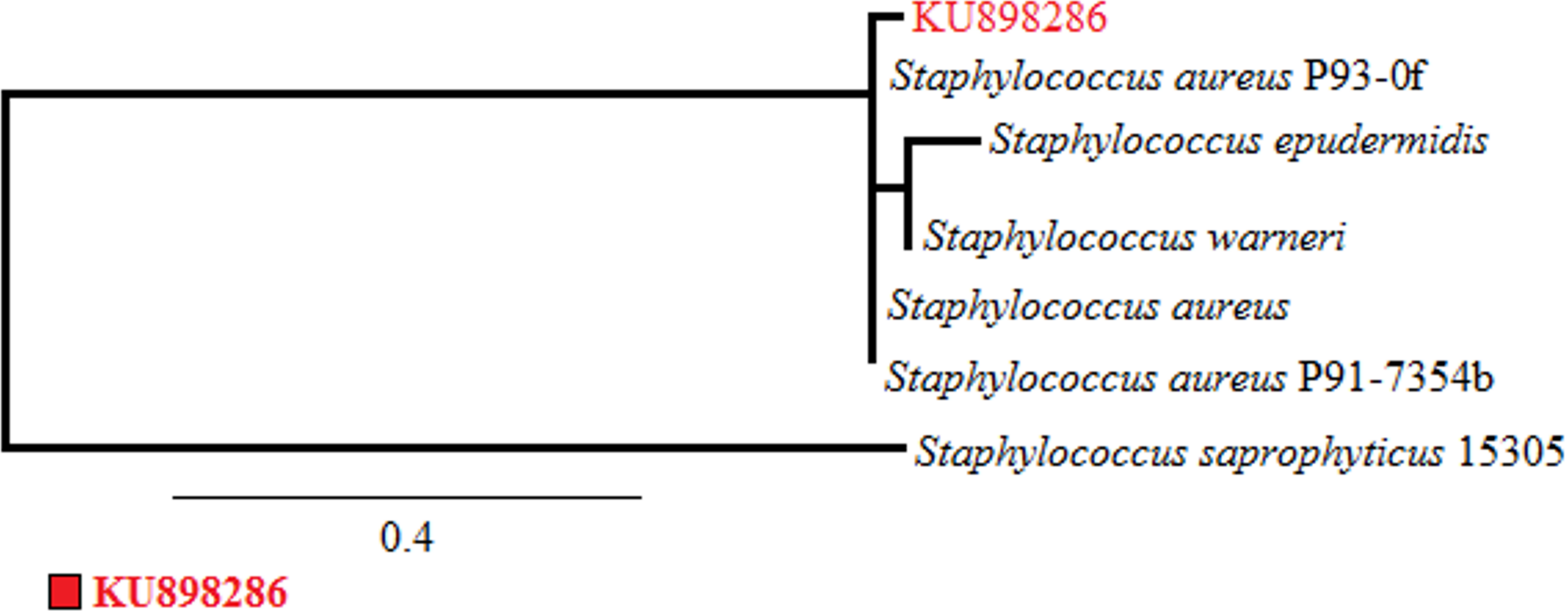
Phylogenetic analysis of the strain S1 (KU898286) following neighbour joining approach. Bootstrap values (100%) as generated from Phylogeny.fr, 2016. Here bar indicates the 0.4 substitution after every nucleotide position.

The strain was observed to be a gram positive that lacked motility. The observed colonies were grape-like having orange-yellow coloured spherical bacteria. The diameter of the bacterial cell was 0.6 μm with colony reaching approx. 2–3 mm on LB agar medium after 24 h of incubation. The strain was observed to be oxidase negative and was negative towards indole. It showed positive tests regarding catalase, DNase production, coagulase reaction and urease, which are in agreement with its resemblance to *Staphylococcus aureus*, according to Bergey’s Manual. The phylogenetic tree (Fig 1; Phylogeny.fr, 2016) constructed by using the neighbour joining approach and 16s rDNA gene NCBI BLAST showed a close match of Strain S1 to *Staphylococcus aureus* (99% resemblance). The sequence was submitted to NCBI Genebank and is available under the accession code KU898286.

### Dye decolourisation by purified azoreductase

**Fig 2:**
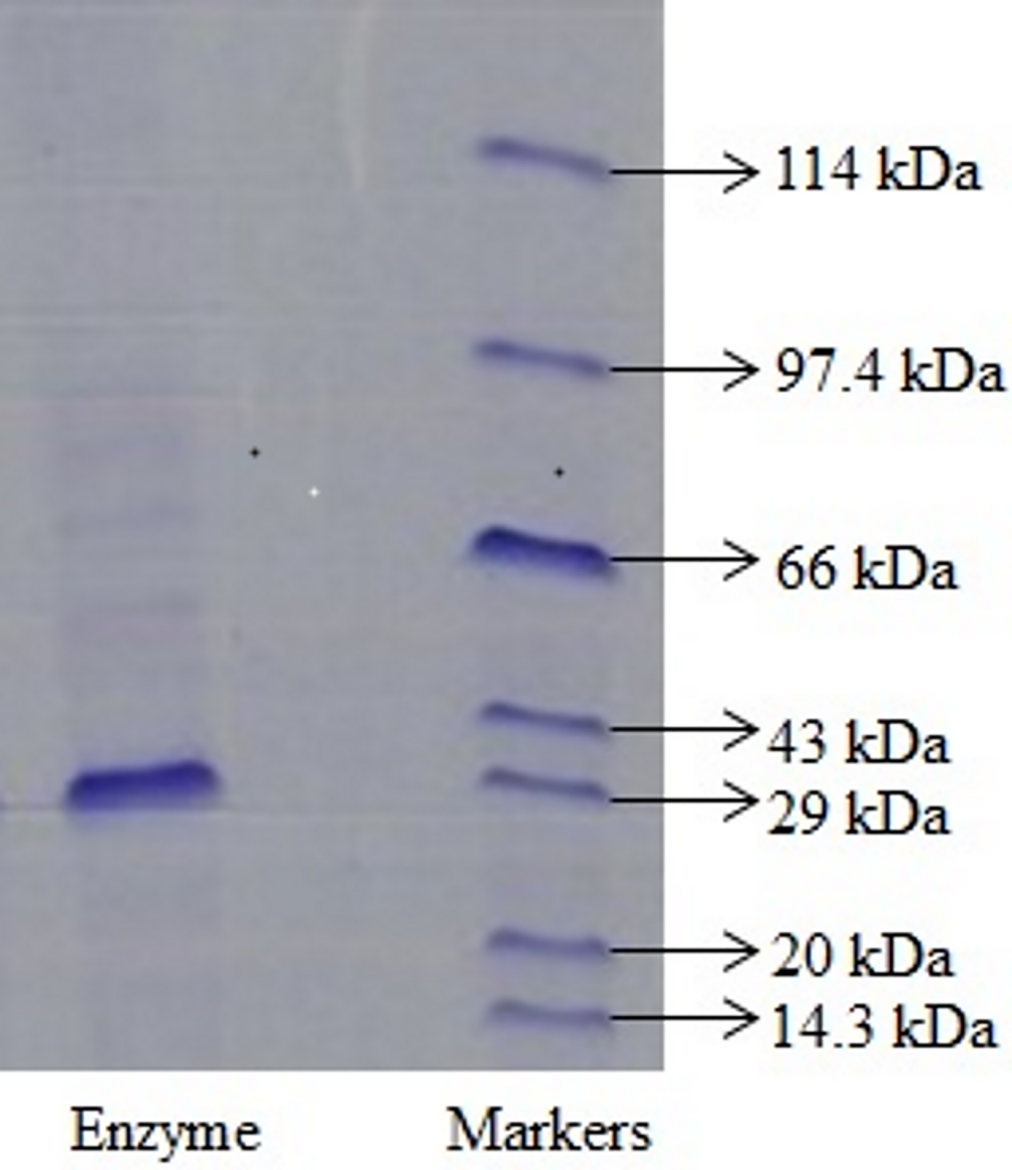
SDS-PAGE of azoreductase (purified) showing a weight of 29 kDa. (10% gel with 0.1% SDS; Lane 2 = 65ng of purified azoreductase).

The crude extracts of *S*. *aureus* (20 g of wet wt) were disrupted and yielded 1142 mg of proteins. About 83% of proteins were lost during the purification step, and the molecular weight of the enzyme was observed to be 29 kDa (Fig 2).

**Fig 3:**
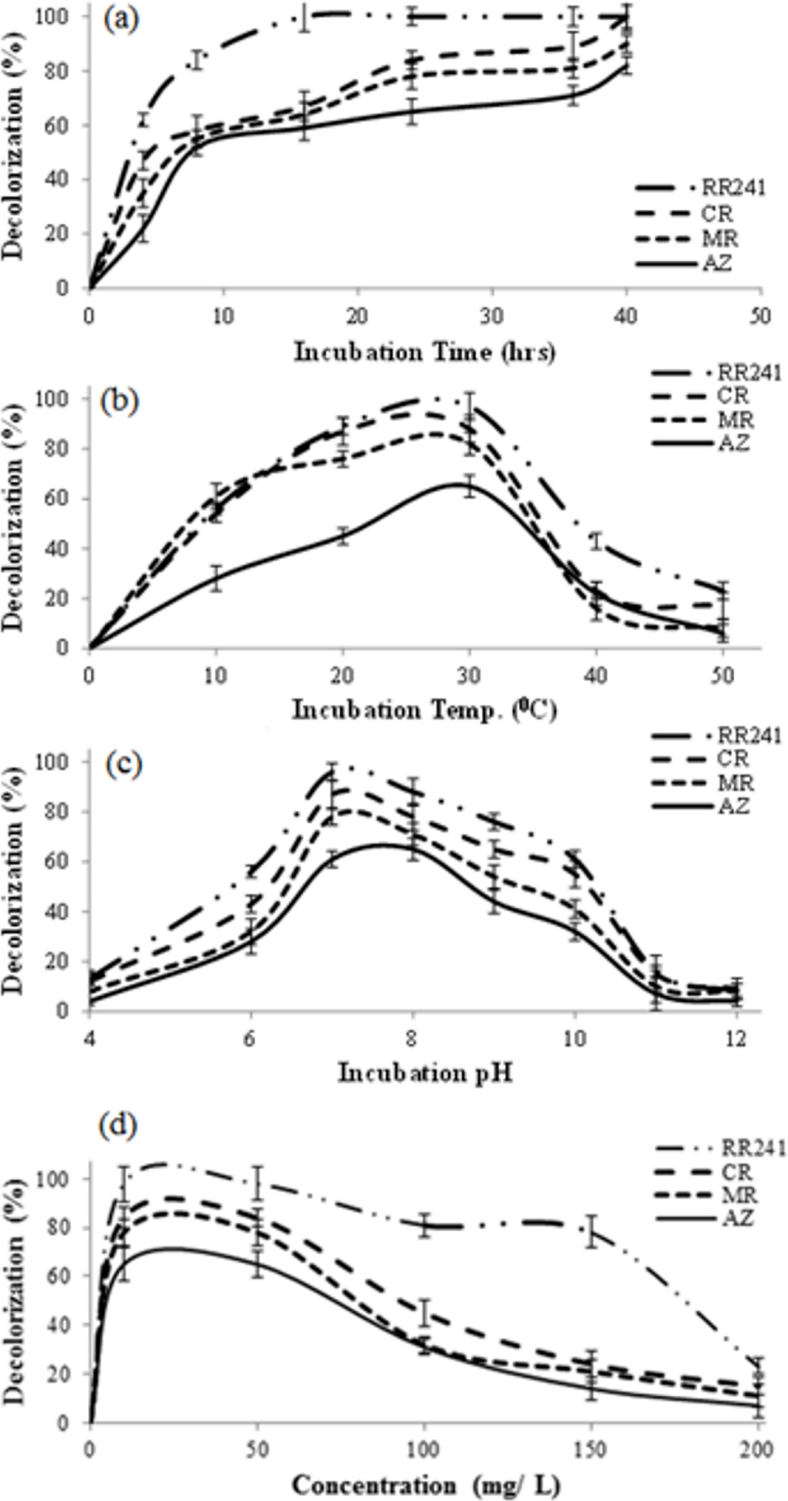
Effect of different conditions on degradation of reactive red 241 (RR241). Congo red (CR), methyl red (MR) and azobenzene (AZ) by azoreductse from bacterial isolate S1 (a) incubation time (0-40hrs) at constant pH 7, temperature of 30°C and concentration of 50mg/L (b) incubation temperature (0–50°C) at constant pH 7, incubation time 16hrs and concentration of 50mg/L (c) pH (4–12) at constant temperature 30°C, incubation time 16hrs and concentration of 50mg/L (d) concentration of substrate at constant temperature 30°C, incubation time 16hrs and pH 7.

The decolorization of azobenzene (AZ) and azo dyes (RR241, methyl red (MR) and congo red (CR) by the purified enzyme was investigated under varying conditions. The isolated strain S1 was capable of surviving at a minimal concentration of RR241 (50 mgL^–1^) and 100% decolourisation efficiency was recorded after 18 h of exposure. Control experiments were performed under identical conditions without azoreductase. As observed the strain S1 was also able to grow on multiple substrates such as AZ, MR and CR (Fig 3A–3D), confirming its ability to reduce azo bonds and sustenance in the culture containing these dyes. As shown in Fig 3A, more than 60% of RR241 was decolourised within the first 5 h of incubation. After 15 h incubation, about 98% of RR241 was degraded, while a complete decolourisation was recorded at about 18h of incubation. The other investigated substrates followed a similar pattern, but at a slower rate.

The enzymatic activities have been reportedly affected by varying medium temperatures; therefore, we investigated the effect of temperature between 0 and 50°C at pH 7.5 on azoreductase activities (Fig 3B). The enzyme showed comparative activity (>40%) towards the substrate over a temperature range from 10 to 20°C. As observed, more than 75% decolourisation was recorded between 20 and 25°C in the presence of RR241, CR and MR. However, the enzyme activity decreased rapidly beyond 30°C, and relatively low enzyme activities were detected at temperatures over 45°C. Interestingly, the enzyme was stable below 30°Cafter 18 h incubation.

The pH activity of the purified enzyme was investigated over a pH range of 4–12. More than 30% of the relative activity was recorded within a pH range from 4.0 to 6.0 (Fig 3C). About 98% decolourisation was achieved for RR241 after 15 h incubation at pH 7.5 and similarly, the maximum enzyme activities were recorded at pH 7.5 for the other substrates (CR, MR and AZ). Steady decreases were noticed beyond pH 7.5 which may be attributed to instability of the enzyme in the alkaline domain. The decolourisation activity of S1 was also evaluated at different enzyme concentration (0–200 mgL^–1^). As shown in Fig 3D, a rapid decolourisation occurred at low enzyme concentrations (10 mgL^–1^) with approximately 78% decolourisation for MR and 98% for RR241 within 5 h of initiation of the reaction. At enzyme concentrations higher than 50 mgL^–1^, a decreasing trend in decolourisation efficiency was observed. Conclusively, these results suggested that 50 mgL^–1^ of the enzyme is required to achieve the highest decolourisation percentage at pH 7.5.

### Kinetic studies of the purified enzyme

**Fig 4:**
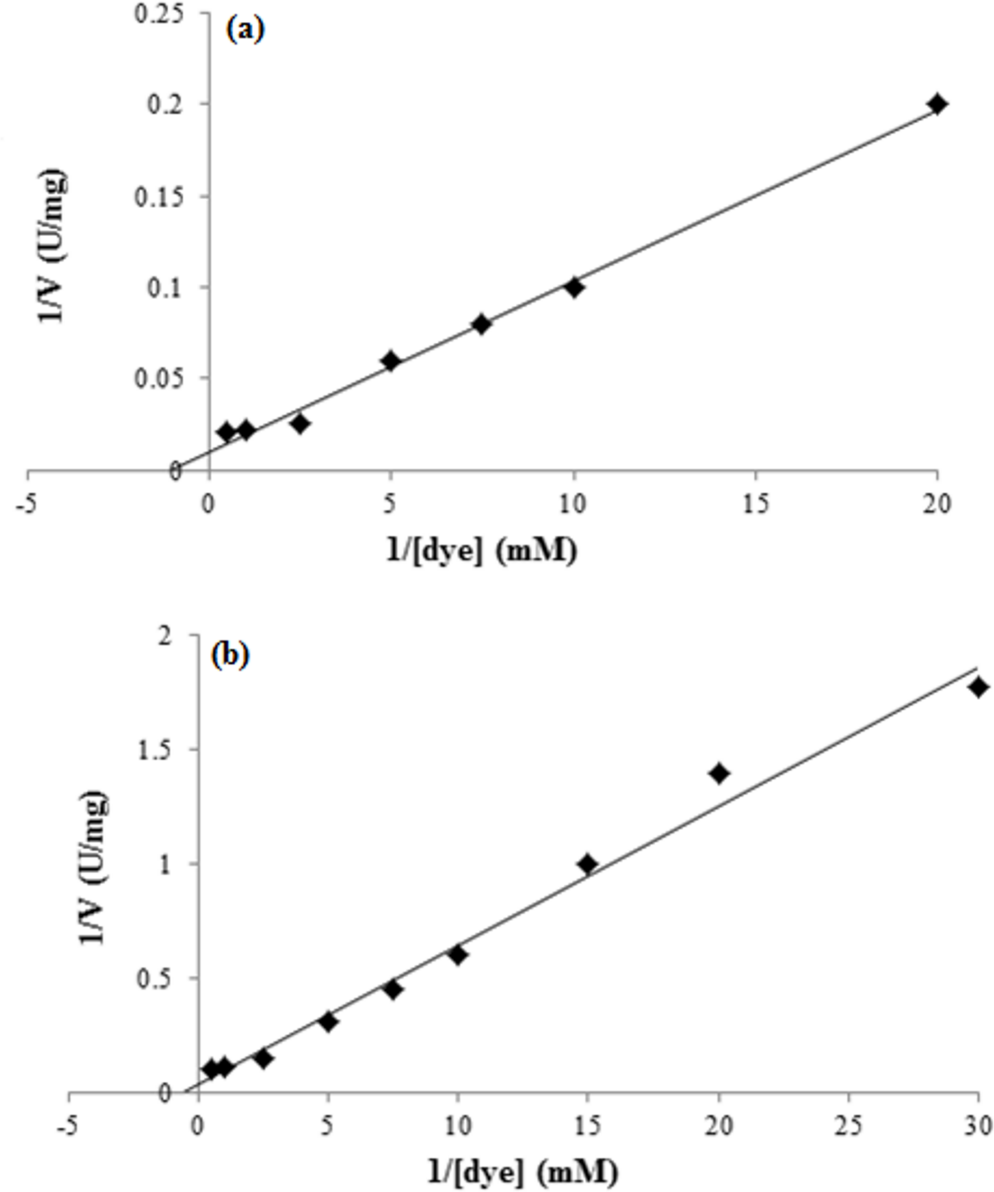
Kinetics of azoreductase as purified from *S*. *aureus* strain KU898286. Figure (4a) represents the velocities observed by varying RR241 dye concentration (0-20mM) in the absence of NADH; Figure (4b) represents the velocities observed by varying NADH concentration (0-30mM) where the RR241 was constant (0.5 mM).

The kinetic properties of the azoreductase were evaluated to observe the oxidation-reduction between the enzyme and substrate in the presence and absence of NADH. The concentration of RR241 or NADH was varied, and the other substrate was kept constant. The maximal velocity (V_*max*_) and Michaelis–Menten constant (K_*m*_) were then obtained from the Lineweaver-Burk plots^21^. As shown in Fig 4A, the concentration of RR241 was varied and at constant NADH (1mM), here the K_*m*_ value was 1.54 mM for 100 Umg^–1^ of V_*max*_. However, in the presence of varied concentration of NADH (Fig 4B), the K_*m*_ value was 1.02 mM for 18.7 Umg^–1^ of V_*max*_ against 0.5 mM RR241. From these analyses, about 4.5 mol of NADH was consumed per unit mol of RR241. The lesser K_*m*_ value for second reactions shows higher substrate affinity and high binding capacity in the presence of NADH.

### Extracellular and intracellular enzymatic activity against RR241

The selected stain S1 exhibited both intracellular and extracellular enzymatic activity. The purified azoreductase had broad spectrum activity at extreme conditions and observed to be oxygen insensitive. Here, two experiments were performed to observe the extracellular and intracellular enzyme activity. The extracellular enzyme was able to decolourise 50 mgL^–1^ of RR241 completely after 24 h of exposure. Interestingly, a complete decolourisation was recorded after 18 h via the intracellular enzymatic activity.

**Fig 5:**
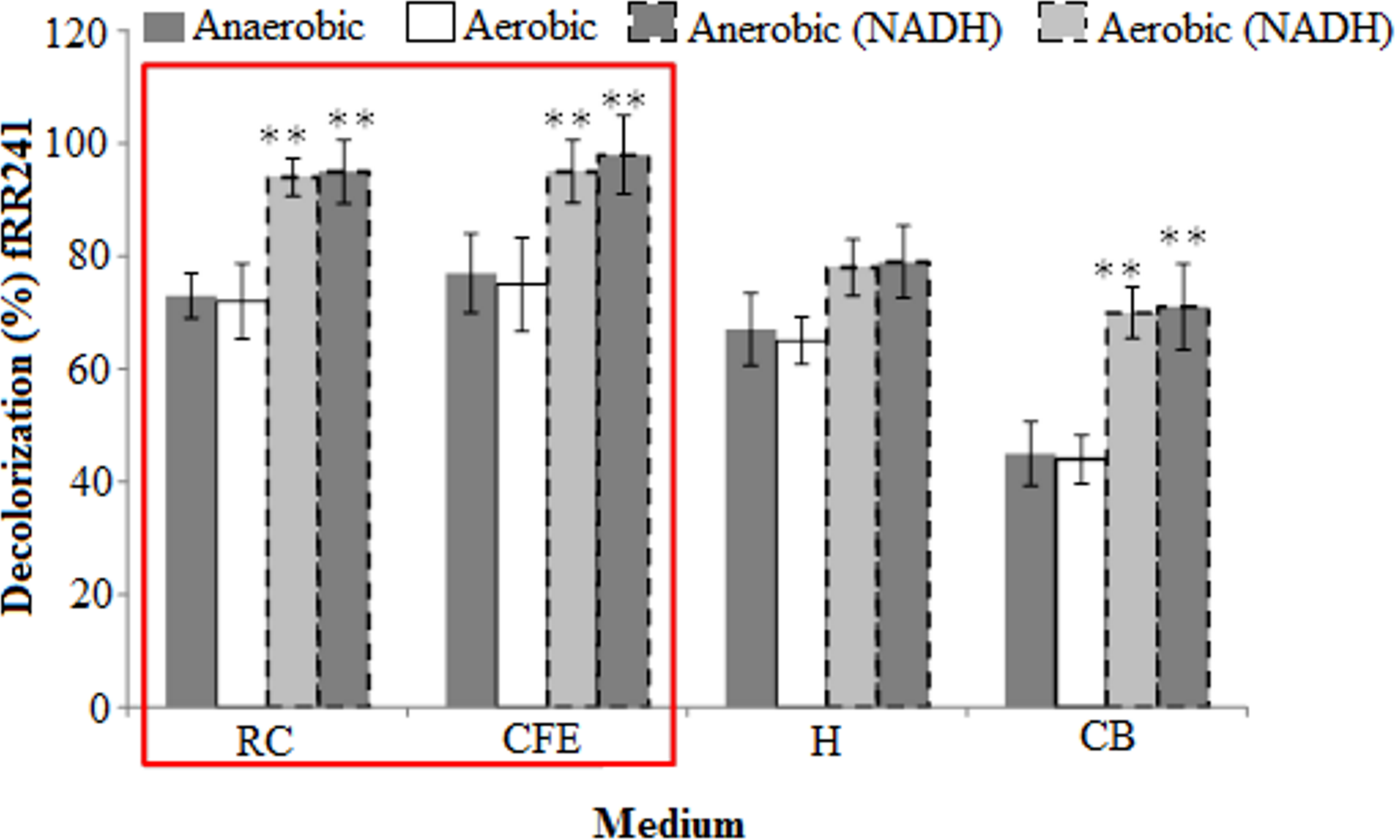
Localisation of activity of azoreductases at constant concentration, temperature, incubation time and pH of RR241. Concentration of RR241 = 50 mg/L; temperature = 30°C; incubation time = 16 hrs and pH = 7. Intracellular activity has been designated by resting cells and cell free extract (red box highlighted portion), whereas extracellular activity was shown by homogenate and cell debris. RC stands for resting cells, CFE stands for cell free extracts, H stands for homogenate and CB stands for cell debris.

The enzyme assay result indicated that the bacterial resting cells and cell-free extract exhibited maximum decolourisation efficiencies for RR241 in the presence of crude NADH under both aerobic and anaerobic conditions. As shown in Fig 5, 93% decolourisation efficiency was recorded under an aerobic condition for cell-free extract and increased slightly to 96% under anaerobic condition with the presence of a same level of NADH. However, less than 80% decolourisation was recorded for homogenate and cell debris. It is important to stress that the azoreductase activity increased as NADH concentration increased and anaerobic condition was required to enhance the azoreductase activity.

### Identification of the reduced products

The UV–vis scan (λ = 400–800 nm) of supernatants showed the decrease in dye intensity with the increase in exposure time to the bacterial cells. Obvious changes in the peaks were detected after 18 h of incubation. It is evidently shown that the bacterial enzymes efficiently degraded the azo bonds into a methane-containing by-product. The FTIR spectrum of the control dye experiment showed S = O stretching vibrations (1048 cm^–1^), asymmetric stretching (2854 cm^–1^) and symmetric stretching at 2926 cm^-1^ representing the C = N bond in the parent compound. The peak at 3422.12 cm^–1^ represents the free NH group as in the parent dye. The C–H stretching was represented by the band at 2924cm^–1^ and the peak at 1578 cm^-1^ is attributed to the–N = N–azo group stretching.

**Fig 6:**
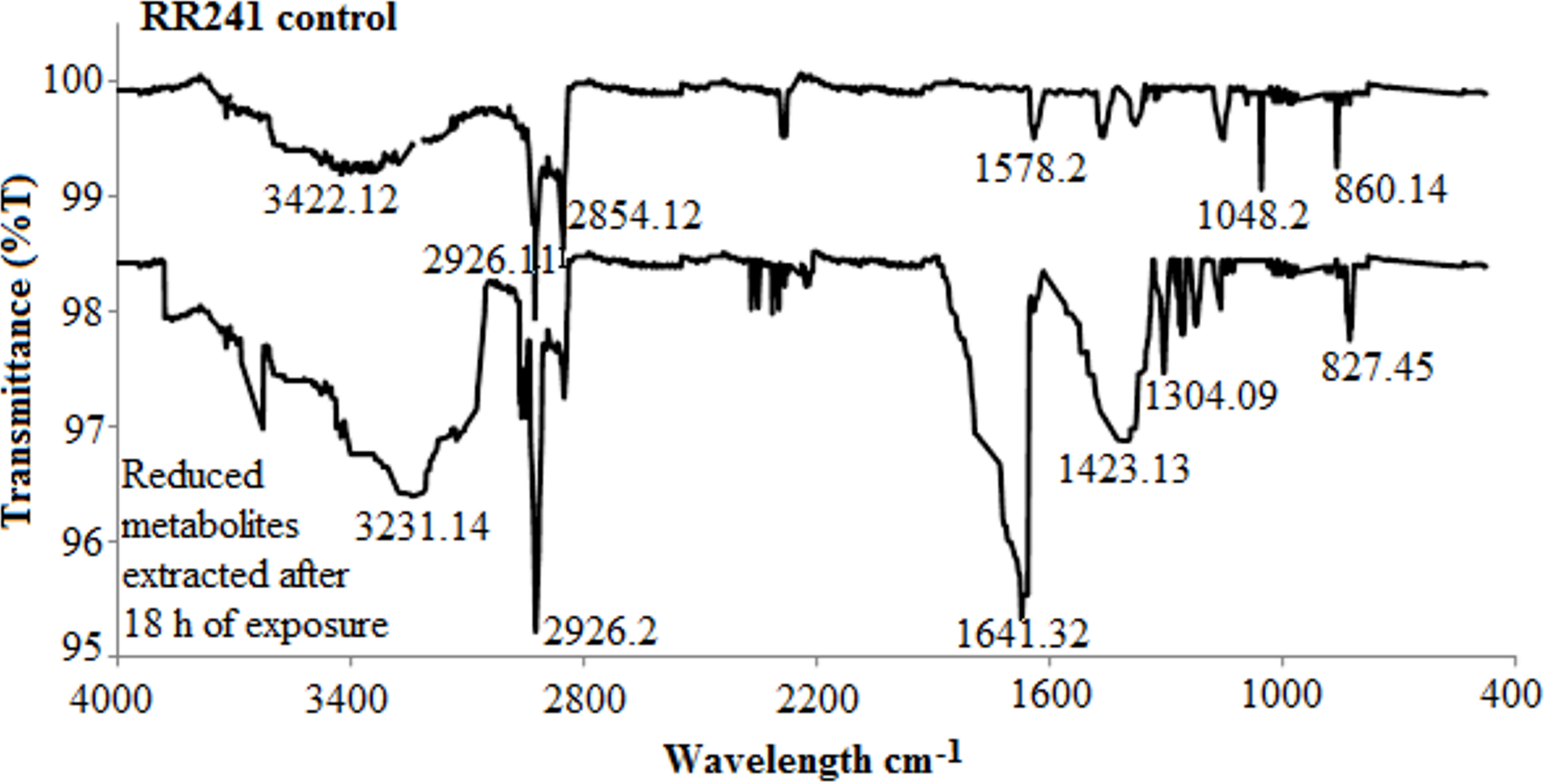
FTIR chromatogram of reactive red 241 and its degradation metabolites.

After 18 h of incubation, the transformation of RR241 into various metabolites was observed by the disappearance and appearance of new IR peaks in the spectrum. For instance, the presence of new peaks around 1304 cm^–1^ represents the C–N vibration due to secondary aromatic amines. The new peaks at 3231, and 1423 cm^–1^ suggest the presence of amide N–H stretching and C–H due to the deformation of CH_2_. The formation of the benzene ring is attributed to the peak at 829 cm^–1^. Significant peaks enhancements were detected at 2926 and 1641 cm^–1^ suggesting the presence of C = N and C = O stretching in the formed metabolites. The absence of a major azo peak at 1578 cm^–1^ (–N = N–) confirmed that RR241 was reductively cleaved (Fig 6).

**Fig 7:**
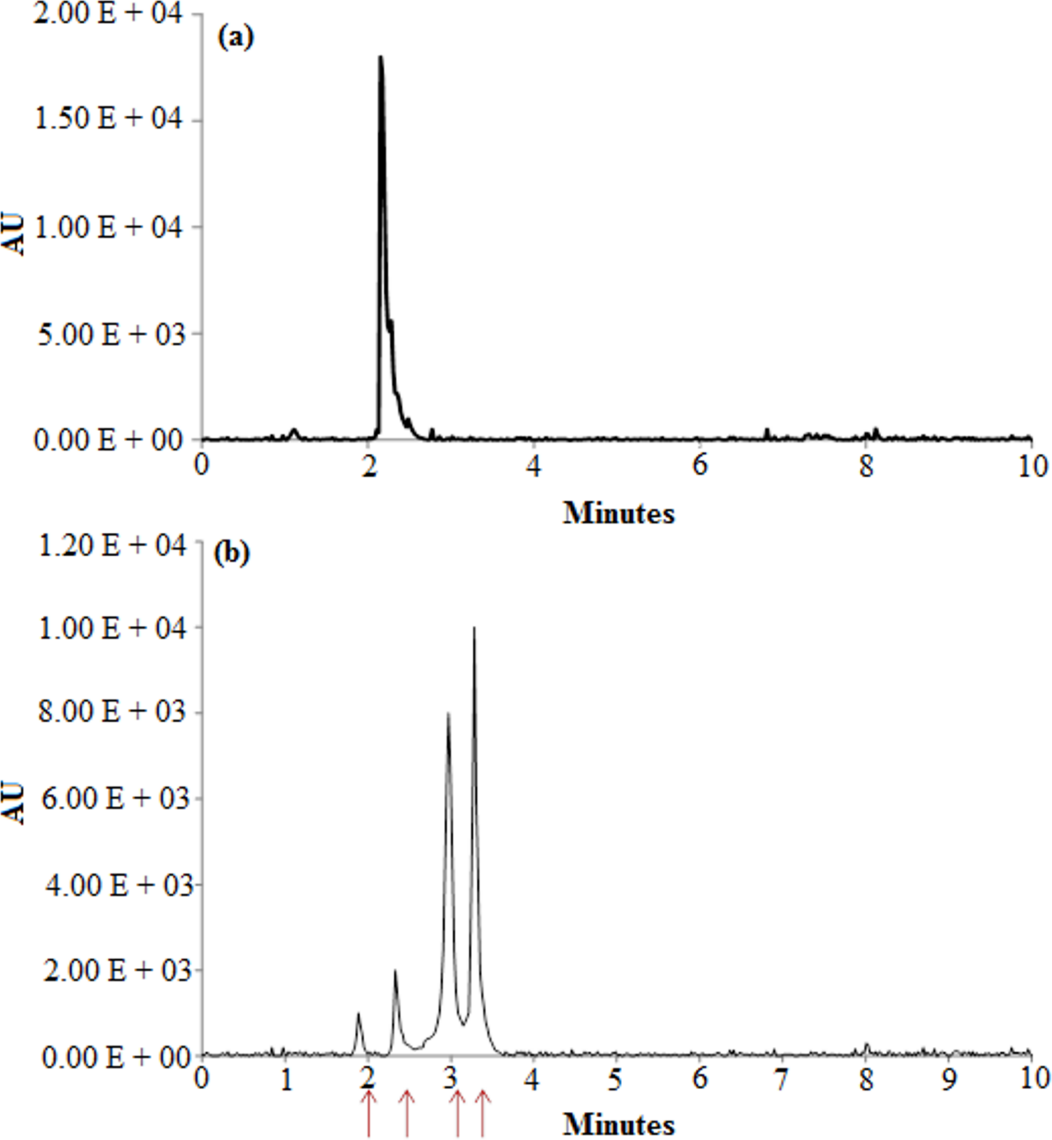
HPLC elution profile. (a) reactive red 241 and (b) its reduced metabolites after 18hrs of exposure to the enzyme (dye conc. was 50mgL^-1^).

HPLC analysis (Fig 7) for the control dye sample before its exposure to the supernatant showed a single peak at retention time of 2.15 min. The analysis of decolourised sample after 18 h of exposure to the bacterial cells revealed two minor peaks at 1.88 min and 2.32 min and major peaks at 2.9 min 3.2 min. Hence, this suggests the conversion of RR241 into various intermediates. The appearance of new minor and major peaks is inferred to be the mineralisation of parent dye.

**Fig 8:**
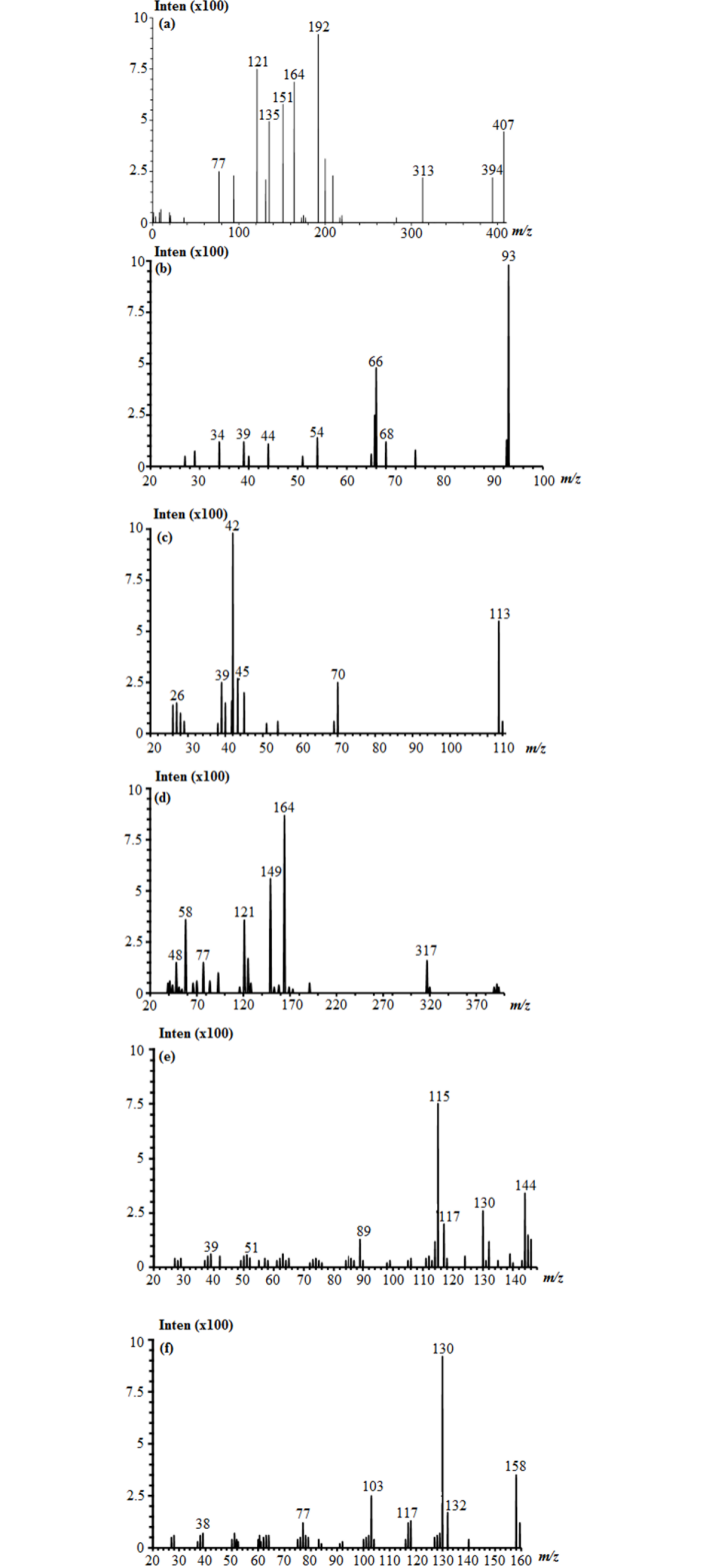
MS spectra of the metabolites through GCMS obtained after 18 hrs of exposure of azoreductase to 50mgL^-1^ dye. MS spectrum of (a) Phenylazo-2-naphthol-5,8-disulfonic acid, (b) aniline, (c) 1,3,5-triazine-2,4-diol, (d) 1-amino-8-naphthol-2,5-disulfonic acid, (e) 2-naphthol and (f) 1-amino-2-naphthol.

**Fig 9:**
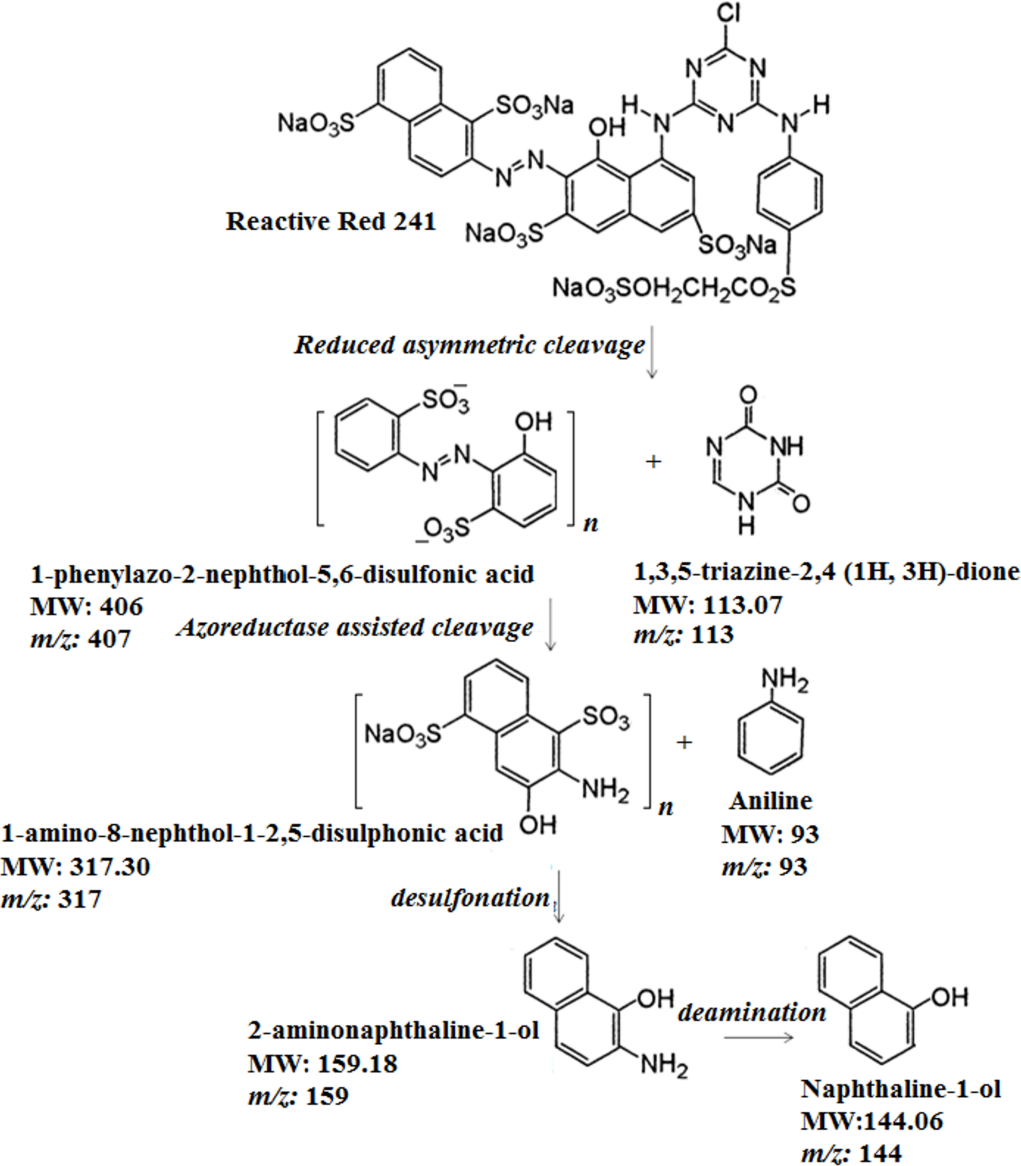
Proposed degradation pathway of reactive red 241 by Staphylococcus sp. KU898286 on the basis of GC-MS traces and their MS interpretation.

GC–MS analysis confirmed the metabolites; however, the exact mechanism is unknown. The chromatograms (Fig 8) revealed the existence of several peaks, (m/z) values and fragmentation pattern corresponding to the probable structures of the detected compounds. The probable mechanism of RR241 degradation is proposed in the present study (Fig 9). RR241 was converted into several intermediates as depicted due to the oxidative cleavage of the N = N of the azo group by the purified enzyme. The formation of 1-phenylazo-2-naphthol 5,8 disuphonic acid (30.33 min) as minor intermediate and 1-amino-8-naphthol as a major intermediate (26.14 min; mass peak 317) was reflected in the mechanism. Fig 9 Proposed degradation pathway of Reactive Red 241 by *Staphylococcus* sp. KU898286 on the basis of GC-MS traces and their MS interpretation.

### Phytotoxicity assay

The parent dye (RR241) and its final degradation product (naphthalen-1-ol) by the isolated *Staphylococcus* sp. KU898286 was assessed for the toxicity. Results predicted that the degradation products are less toxic compared to the parent compound (Table 1). No germination was recorded in case of A. *thaliana*, whereas only 2% germination rate was recorded for *S*. *vulgare*, when irrigated with RR241 containing water. Furthermore, the growth of the radicle and plumule of the seedlings irrigated with RR241 mixed water was significantly (*P*<0.001) effected as compared to the control (irrigated with tap water). On the contrary, no significant difference was observed concerning the plumule and radicle growth of the seedlings irrigated with the breakdown products of RR241 to that of control.

**Table 1:** Phtotoxicity assessment of reactive red 241 and its degradation product.

	*Sorghum vulgare*			*Arabidopsis thaliana*		
*Parameters*	*Tap water*	*RR241*	^*1*^*Metabolites*	*Tap water*	*RR241*	^*1*^*Metabolites*
Germination%	100	2***	90	100	0***	80
Radicle (cm)	7.1±1.21	0***	5.41±0.88	4.01±0.67	0***	2.87±0.51
Plumule (cm)	7.2±1.26	0***	6.44±1.01	4.75±0.58	0***	3.77±0.8

Values are the mean with SE (±)Concentrations of RR241 and its metabolites were 500mg/L.

^*1*^*Metabolites* represents the degradation products of RR241

*P<0.05 and

***P<0.001 as values compared to controlled by one-way ANOVA

## Discussion

The present study suggested the isolation of a novel strain of *Staphylococcus* sp. that was capable of reducing reactive red 241 completely to a harmless by products after 18 hrs of exposure. The acclimatized culture was tested against different substrates, but have highest activity against RR241 (almost 98% of dye was consumed after 15hrs of exposure), which might be linked to the substrate specificity/recognition for the dye by the strain [4, 8]. Similar observations have been reported on many azoreductases with an optimum pH range of 6.5–7.4 [1, 16, 17]. It is inferred that the synergistic action of these oxido-reductase enzymes caused the degradation of RR241. Specifically, the sulphate bonds at the terminal of RR241 can easily bind to the bacterial enzymes; hence, resulting in reduction and subsequent cleavage of the azo bonds [4, 8]. This might be one of the reasons that RR241 was easily available to the isolated species compared to the other substrates.

Furthermore, the enzyme can utilize NADH for its activity as evident from Fig 4A and 4B, where lesser K_*m*_ indicates the high substrate binding and affinity. Briefly, electrons provided by the FAD reduction from FADH_2_ in the presence of NADH contributed towards the reduction of sulfonated azo dyes [18]. That might be the main cause for speeding up the reaction in the presence of NADH. The enzyme activities in this study were consistent with those reported by Wang et al. [16]. In their study, they have observed the decolourisation of reactive black azo dye by NADH-dependent azoreductase derived from *Bacillus sp*. Though, extensive studies have shown the application of lignolytic enzymes of fungul origin such as *B*. *adusta* and *P*. *chrysosporium* [19, 20] having high applications against polymeric dyes. But, recently it has been explored that azoreducatases are the key enzymes to catalyse the azo dye degradation through the reductive cleavage. This enzyme utilizes NADH for its efficient activity. From the analysis of current study (Fig 4B), it can be observed that about 4.5 mol of NADH were consumed per each mol of RR241. HPLC and FTIR analysis confirmed the proposed degradation pathway (Fig 8), which suggests the conversion of azo linkages through the formation of intermediates (such as phenyl radicals). The end product has been observed to be a 2-naphthol with the molecular weight of 144.

From the result of this research, we can propose that toxic RR241 can be degraded by *Staphylococcus* KU898286 efficiently to a less toxic product (2-naphthol). From the study, we have concluded that the isolated strain of *Staphaloccocus* (KU898286) can ably reduce/metabolize the toxic azo dye RR241 to a non-toxic product. The electron with-drawing nature of azo dyes and their preference for reductive metabolism has been evident from the present study. Moreover, the presence of NADH increased the rate of digestion of azo dyes by the bacterial cytoplasmic enzymes, leading to a complete degradation of the azo dye.

## Abbreviations

AZ: Azo benzene
BSA: Bovine serum albumin
CR: Congo red
DNA: Deoxyribonucleic acid
DSM: Difco Sporulation medium
FTIR: Fourier transformation infra-red spectrophotometry
GC-MS: Gas chromatography-Mass spectroscopy
HPLC: High pressure liquid chromatography
LB: Luria-Bertani broth
MR: Methyl red
MSM: Murashige and Skoog medium
NADH: Nicotinamide adenine dinucleotide
NMR: Nuclear magnetic resonance
OD: Optical density
RNA: Ribonucleic acid
SDS-PAGE: Sodium dodecyl sulphate-polyacryl amide gel electrophoresis
